# Effects of a lymphedema prevention program based on the theory of knowledge–attitude–practice on postoperative breast cancer patients: A randomized clinical trial

**DOI:** 10.1002/cam4.6171

**Published:** 2023-06-17

**Authors:** Bohui Shi, Zihan Lin, Xiaowei Shi, Pingli Guo, Wen Wang, Xin Qi, Can Zhou, Huifang Zhang, Xiaona Liu, Aili Iv

**Affiliations:** ^1^ Department of Breast Surgery, The First Affiliated Hospital of Xi'an Jiaotong University Xi'an Shaanxi China; ^2^ School of Nursing Xi'an Jiaotong University Health Science Center Xi'an Shaanxi China; ^3^ Department of Paediatrics The First Affiliated Hospital of Xi'an Jiaotong University Xi'an Shaanxi China; ^4^ Infectious Department The First Affiliated Hospital of Xi'an Jiaotong University Xi'an Shaanxi China; ^5^ Precision Medicine Center The First Affiliated Hospital of Xi'an Jiaotong University Xi'an Shaanxi China

**Keywords:** arm disability, breast cancer, hand strength, knowledge–attitude–practice, lymphedema, quality of life

## Abstract

**Background:**

Breast cancer‐related lymphedema (BCRL) is one of the common postoperative complications that severely affects the functions of the arm and quality of life. Since lymphedema is difficult to treat and prone to recurrence, early prevention of lymphedema is crucial.

**Methods:**

Patient diagnosed with breast cancer (*N* = 108) were randomized to the intervention (*n* = 52) or control group (*n* = 56). In the intervention group, patients were provided the lymphedema prevention program based on the theory of knowledge–attitude–practice during the perioperative period and the first three chemotherapy sessions (mainly includes health education, seminars, knowledge manuals, sports guidance, peer education, and WeChat group).The limb volume, handgrip strength, arm function, and quality of life were measured in all patients at the baseline, 9 weeks (T1), and 18 weeks (T2) after surgery.

**Results:**

The incidence of lymphedema in the Intervention group was numerically lower than in the control group after implementing the lymphedema prevention program, but the difference was not statistically significant (T1: 1.9% vs. 3.8%, *p* = 1.000; T2: 3.6% vs. 7.1%, *p* = 0.744). However, compared with the control group, the intervention group showed there was less deterioration in handgrip strength (T1 [*t* = −2.512, *p* < 0.05] and T2 [*t* = −2.538, *p* < 0.05]), improved postoperative upper limb dysfunction (T1 [*t* = 3.087, *p* < 0.05] and T2 [*t* = 5.399, *p* < 0.05]) and less deterioration in quality of life (T1 [*p* < 0.05] and T2 [*p* < 0.05]).

**Conclusion:**

Although the investigated lymphedema prevention program improved arm function and quality of life, it did not reduce the incidence of lymphedema in postoperative breast cancer patients.

## 1 INTRODUCTION

Epidemiological investigations reported that there were 19.3 million new cancer cases worldwide in 2020, of which female breast cancer accounted for 11.7% and was ranked first in cancer incidence among women.^1^ Surgery is one of the most common treatments for breast cancer but is often associated with complications such as wound bleeding, infection, subcutaneous hydrops, flap necrosis, limb dysfunction, and breast cancer‐related lymphedema (BCRL).^2^, ^3^ BCRL can lead to physical and psychological problems such as limb dysfunction, hypotonia, appearance‐related issues, anxiety and depression in breast cancer patients, thereby seriously affecting postoperative rehabilitation and quality of life.^4^ According to relevant reports, BCRL may appear soon after surgery or occur late during recovery and has a prevalence of approximately 21.4% (95% CI: 14.9%–29.8%).^5^ At presently, physical and drug therapy or surgery can improve lymphedema, but many patients cannot adhere to treatment due to its long course, high cost and unsustainable effects.^6^ BCRL has been recognized as a difficult complication to treat during postoperative recovery and requires medical professionals' attention.

Since BCRL is difficult to cure once it occurs, increasing patient awareness and education about the risk of lymphedema and focusing on prevention and early lymphedema intervention are even more necessary to improve the quality of life and survival of breast cancer patients. The initial manifestations of BCRL, such as numbness and soreness, are easily overlooked by patients. A study from China used a self‐designed postoperative lymphedema prevention knowledge questionnaire to survey breast cancer patients and reported that they had poor knowledge of BCRL,^7^ which was significantly lower than the international median lymphedema knowledge rate.^8^ Some researchers interviewed BCRL patients and health professionals and found that healthcare professionals needed to provide more educational programs and comprehensive support to enhance patient self‐management of lymphedema.^9^

Whitworth et al.'s systematic review showed that prospective BCRL surveillance combined with early detection and intervention could reduce the incidence of chronic BCRL.^10^ A systematic review of research on lymphedema self‐management summarized ten categories of self‐management and found that whole‐body exercise, second‐stage CDT, including compression garments, exercise, careful skin care, and compression bandages, might be effective in preventing BCRL.^11^ A summary of 24 studies by Fu et al. found that breast cancer survivors faced challenges in lymphedema self‐management and needed to reestablish a new role balance as well as internal and external resources to regulate negative emotions.^12^ Although most of the related studies have assessed the effectiveness of a single intervention, there is a lack of systematic lymphedema prevention programs for postoperative breast cancer patients.

Knowledge–attitude–practice (KAP) is currently used in patient rehabilitation guidance and education.^13^ Some cross‐sectional studies were conducted on KAP to assess people's acquisition of research‐related knowledge, attitudes, and practices.^14^, ^15^ Wang et al. reported that interventions using KAP‐based rehabilitation education in patients with lumbar disc herniation increased patients' awareness on rehabilitation and promoted positive rehabilitation behaviors.^16^ It can be seen that this theory can promote the mastery of patients' health knowledge, improve patients' awareness of rehabilitation, and take more active actions to promote health.^13^ However, no KAP‐based interventions for lymphedema prevention have been retrieved. Moreover, the effectiveness of current intervention studies on the prevention of lymphedema after breast cancer surgery is inconsistent, which requires further verification.This present study intended to develop a prevention program for upper limb lymphedema in postoperative breast cancer patients based on the KAP theory and combined with the best evidence for upper limb lymphedema prevention for clinical application to: (1). promote patients' ability and adoption of positive rehabilitation behaviors to reduce the incidence of upper limb lymphedema; (2). improve handgrip strength and function of the affected upper limb; and (3). improve patients' quality of life.

## 2 METHODS

### 2.1 Design

This randomized, controlled trial was conducted at a tertiary public hospital in Xi'an, Shaanxi Province, China. Patients who were diagnosed with breast cancer by pathologic puncture between March 2020 and November 2020 were recruited. Patients were enrolled before surgery. The study protocol was based on the CONSORT 2010 checklist and was registered retrospectively on clinicaltrials.gov (NCT05595330).

### 2.2 Sample

#### 2.2.1 Inclusion criteria

The study inclusion criteria were as follows: (1) female patients aged ≥18 years old; (2) pathological puncture confirming unilateral breast cancer without recurrence and metastasis; (3) clinically staged as TNM stage I‐III; (4) plan to perform surgery and ≥6 cycles of postoperative chemotherapy; and (5) conscious and aware of their disease condition, with no cognitive impairment or communication problems.

#### 2.2.2 Exclusion criteria

The study exclusion criteria were as follows: (1) patients with cancer other than breast cancer; (2) a history of arm or neck trauma, infection, or surgery; (3) the presence of serious diseases such as cardiovascular, cerebrovascular, liver, and kidney; (4) had upper limb disability, or the affected limb had edema before surgery; (5) thrombus in the blood vessels of the affected limb; and (6) patients who received neoadjuvant chemotherapy.

#### 2.2.3 Power analysis

This study used the PASS v15.0 software to calculate the sample size, using the two‐sided test, *α* = 0.05 and 1‐*β* = 0.8. The reported incidence of lymphedema varies among different countries, such as 8.4% (95% CI: 15.0%–29.8%) in the UK and 21.5% (95% CI: 5.1%–13.4%) in Australasia.^5^ In China, one study showed that the incidence of lymphedema in the intervention group and the control group was 2.5% versus 22.5%, and another study found that it was 5.93% versus 33.07%. After considering the above data and inviting lymphedema experts to discuss,^17^, ^18^ P1 was taken as 30% and P2 as 5%. The final calculation yielded N1 = N2 = 44, considering a 20% loss to follow‐up after surgery, and the final total sample size was set at 106 cases, with 53 cases in the intervention group and 53 cases in the control group. In this study, a total of 108 patients completed the baseline survey, comprising 56 in the control group and 52 in the intervention group. Finally, 50 patients in the control group and 47 patients in the intervention group completed the study.

### 2.3 Randomization

The unit admitting breast cancer was divided into four medical groups, the staffs were adequately trained, and the surgeries were performed in a homogeneous manner. As each medical group managed a fixed number of patients and wards, patients in different medical groups were treated in other wards. To avoid contamination between the intervention and control groups, the study was conducted in regional randomized groups based on departmental medical groups. Four labels were placed in four opaque envelopes, two with 0 and the other with 1. Four physicians representing their respective groups drew the envelopes, and patients enrolled in the two medical groups with “0” were designated as the control group and the other two groups as the intervention group.

### 2.4 Procedure

#### 2.4.1 Intervention and usual care

In the pre‐study phase, the research team conducted a best‐evidence summary of lymphedema prevention strategies for postoperative breast cancer patients.^19^ Forty‐eight items were summarized, including exercise regimen, skin protection, air travel, weight control, physical therapy, self‐monitoring, and daily life precautions. Then, a postoperative lymphedema prevention program for breast cancer was developed based on the KAP theoretical model combined with the best‐evidence summary of lymphedema prevention strategies. Afterward, 15 specialists in medicine, rehabilitation, and care were contacted for two rounds of correspondence; the final prevention program was determined.

The individuals in control group were given the following interventions: routine perioperative education, and nursing and follow‐up in perioperative period; side effects nursing and health education during chemotherapy. Intervention group followed the basic steps of KAP: (1) Knowledge education during the perioperative period: On the basis of routine perioperative health education, lymphedema specialized knowledge was transmitted to patients through many methods, including specialized lectures, symposiums, the distribution of paper materials, WeChat public accounts and WeChat groups. Besides, diary cards were issued to patients to record the compliance of lymphedema prevention behavior, and telephone follow‐up was conducted to solve patients' questions; (2) promote a proactive lymphedema prevention attitude during the patient's first admission to chemotherapy: We introduced the harm of lymphedema, promoted patients to understand their own behaviors prone to lymphedema through diary cards and seminars, guided patients to do functional exercises of affected limbs, and invited rehabilitation volunteers after breast cancer surgery to share their rehabilitation experience, so as to enhance patients' confidence; (3) during the second admission for chemotherapy, the patient was urged to implement the lymphedema prevention method: Strengthen the knowledge of lymphedema for patients, guide patients to learn lymphedema evaluation methods, and urge patients to implement lymphedema prevention measures through on‐site guidance, diary card and telephone follow‐up; (4) the knowledge, attitude, and behavior of lymphedema were assessed for patients at the third admission for chemotherapy, and targeted guidance was given. The specific implementation process is shown in Appendix 1.

#### 2.4.2 Data collection

Data were uniformly collected, and the collectors were blinded to the grouping of the patients. Before the intervention, they explained the whole process of the prevention program, its purpose and its significance to the patients in detail. Then, they asked the patients to fill out an informed consent form, informing them of their right to withdraw from the trial at any time.

A total of 3 time periods of patient data were collected for the study. Phase 1 (T0) collected baseline information from patients before the intervention; phase 2 (T1) was the patient's 3rd postoperative chemotherapy visit (9 weeks after surgery); and phase 3 (T2) was the 6th chemotherapy visit (18 weeks after surgery).

#### 2.4.3 Measures

##### Demographic information

The subject group prepared the questionnaire after literature search and discussion, which included age, BMI, marital status, education level, occupation, monthly income, affected extremity, disease stage, number of axillary lymph nodes extracted, time of drainage tube removal, and operation type. The data collectors collected demographic information, DASH and FACT‐B 4.0 using a unified guidance language. In this study, the patients completed the questionnaire independently as far as possible. For those who could not fill in the form in person, the collector filled the form truthfully according to the patient's descriptions.

##### Limb volume

An inelastic flexible soft ruler was used to measure the circumference at five positions on the flat wrist crease, 0 cm, 10 cm, 20 cm, 30 cm, and 40 cm on the wrist crease. The upper limb was divided into four truncated cones, and the measured value was accurate to 0.1 cm. The upper arm volume was calculated using the formula:

$$V=h\left(C1^{2}+C2^{2}+C1\times C1\right)÷2\pi$$

where each *h* represents 10 cm, *C*1 and *C*2 represent the circumferences next to the two measurement points, and then, the limb volumes were added up at several positions to obtain the upper arm volume.^20^

According to the International Society of Lymphoma, a volume difference of 10% or more was defined as lymphedema, < 20% as mild lymphedema, 20%–40% as moderate lymphedema, and >40% as severe lymphedema.^21^

##### Handgrip strength

Hand grip strength was measured by an electronic grip strength meter in kilograms. Briefly, the patients were instructed to stand straight with the arm down, and the affected hand grasped the grip strength meter with force. Two measurements were taken, and the average value was used.

##### Range of motion

The range of motion (ROM) of the upper limb of breast cancer patients was measured using a circular goniometer (0°–360°). The patients were asked to sit on a bench, and the rehabilitation technician measured the upper limb mobility in six directions: flexion, extension, abduction, adduction, internal rotation, and external rotation.^22^

##### Arm disability

Disabilities of the Arm, Shoulder, and Hand (DASH) is an 11‐item questionnaire developed by Beaton and was used in this study to assess upper‐limb musculoskeletal disorders.^23^ The DASH scores ranged from 0 to 100, with higher scores indicating higher levels of disability. The overall Cronbach's *α* coefficient of the Chinese version of the questionnaire was 0.911, and the intra‐group correlation coefficient was 0.882.^24^

##### Quality of life

Quality of life was evaluated using the Functional Assessment of Cancer Therapy‐Breast (FACT‐B, version 4), developed by Cella et al.^25^ The questionnaire had 36 items classified into five dimensions, and all items were measured using a 5‐point Likert scale, with higher scores indicating better quality of life. It was translated into Chinese by Wan et al., and the Cronbach's *α* for each sub‐scale was 0.61–0.84.^26^

### 2.5 Data analysis

Data analysis was performed using the SPSS 25.0 software. All primary and secondary end points were evaluated in the modified intention‐to‐treat analysis. If the measurement data were normally distributed, it was expressed as mean ± standard deviation ($\bar{x}$ ± SD), and two independent samples *t*‐test was used; otherwise, the median and quartile range was used to express [M(Q)] and the Mann–Whitney U‐test. Count data are represented as frequency and composition ratio using the chi‐square test. The Mann–Whitney U‐test was used for ordinal data. ANOVA was used to analyze the time effect, group effect, and interaction effect of group × time of the research indicators in the two groups of patients, and *η*P^2^ was used to measure the effect size of the intervention.

### 2.6 Ethics considerations

The study was conducted under the Declaration of Helsinki, and written informed consent was obtained from all study patients. The study was strictly voluntary, confidential, fair, beneficial, and safe, and was approved by a university clinical research ethics committee (ethical review number: 2020‐1372).

## 3 RESULTS

### 3.1 Participant characteristics

**FIGURE 1:**
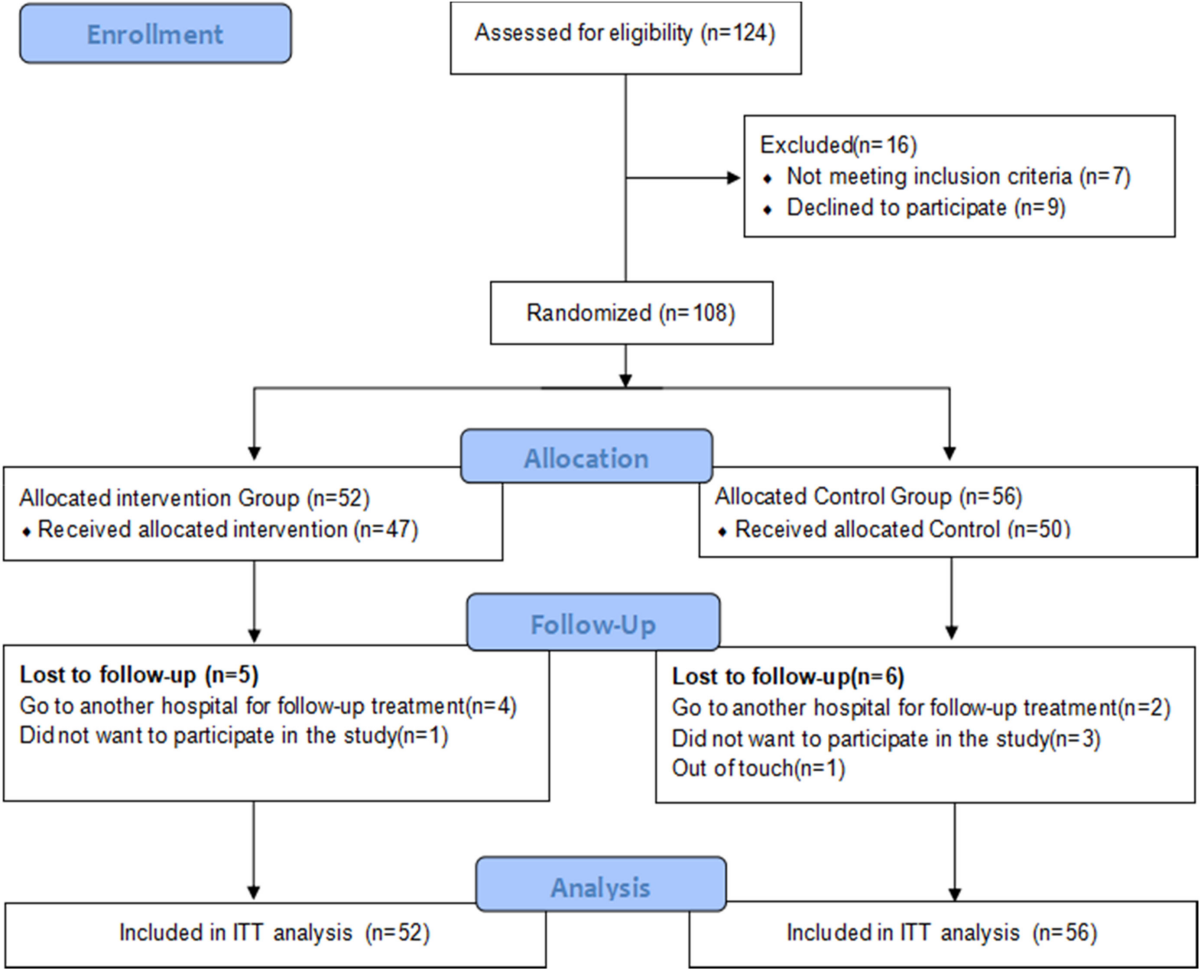
Flowchart of the study process.

In this study, a total of 108 patients completed the baseline survey (T0), comprising 56 in the control group and 52 in the intervention group. A total of 11 patients were lost to follow‐up during the study (6 in the control group and 5 in the intervention group). Lastly, a total of 97 patients completed the study (50 in the control group and 47 in the intervention group; Figure 1).

Statistics of the basic data and lymphedema‐related conditions of the two groups showed that all indicators were well‐balanced (*p* > 0.05) between the groups (Table 1).

**TABLE 1:** Socio‐demographic and clinical characteristics at baseline after group allocation.

Characteristics	Total (*n* = 108)	Intervention group (*n* = 52)	Control group (*n* = 56)	*t*/*Z*/*χ* ^*2*^	*p*
Age ($\bar{x}$ ± SD)	50.32 ± 9.70	49.58 ± 11.03	51.02 ± 8.33	0.762	0.448^a^
BMI ($\bar{x}$ ± SD)	23.48 ± 2.75	23.38 ± 2.66	23.57 ± 2.66	0.363	0.717^a^
Marital status (%)					
Married	102 (94.4)	49 (94.2)	53 (94.6)	1.601	0.898^c^
Never married	1 (0.9)	1 (1.9)	0 (0.0)		
Divorced	3 (2.8)	1 (1.9)	2 (3.6)		
Widowed	2 (1.9)	1 (1.9)	1 (1.8)		
Education level (%)					
Junior high school or below	47 (43.5)	23 (44.2)	24 (42.9)	−0.069	0.945^b^
High school or technical school	28 (25.9)	11 (25.0)	15 (26.8)		
College or above	33 (30.6)	16 (30.8)	17 (30.4)		
Occupation (%)					
Enterprise personnel	189 (17.6)	11 (21.2)	8 (14.3)	2.746	0.739^c^
Peasantry	32 (29.6)	14 (26.9)	18 (32.1)		
Merchant	16 (14.8)	9 (17.3)	7 (12.5)		
Technicist	15 (13.9)	5(9.6)	10(17.9)		
Unemployed	14 (13.0)	7 (13.5)	7 (12.5)		
Others	12 (11.1)	6 (11.5)	6 (10.7)		
Monthly income (Chinese RMB) (%)					
<1500	18 (17.6)	9 (17.3)	9 (16.1)	−0.327	0.744^b^
1500–3000	40 (37.0)	19 (36.5)	21 (37.5)		
3001–5000	26 (24.1)	14 (26.9)	12 (21.4)		
>5000	24 (22.0)	10 (19.2)	14 (35.0)		
Affected extremity (%)					
Left	50 (46.3)	22 (42.3)	28 (50.0)	0.642	0.423^c^
Right	58 (53.7)	30 (57.7)	28 (50.0)		
TNM stage (%)					
I	45 (41.7)	25 (48.1)	20 (35.7)	−1.053	0.293^b^
II	46(42.6)	19 (36.5)	27 (48.2)		
III	17 (15.7)	8 (15.4)	9 (16.1)		
Operation type (%)					
Mastectomy	84 (77.8)	40 (76.9)	44 (78.6)	0.942	0.676^c^
Conserving surgery	18 (16.6)	8 (15.4)	10 (17.9)		
Mastectomy and rebuild	6 (5.6)	4 (7.7)	2 (3.6)		
Number of axillary lymph nodes extracted [M(Q)]	10.19 (3.00, 18.75)	11.84 (3.00, 20.75)	8.64 (3.00, 16.00)	−0.851	0.064^b^
Time of drainage tube extraction (day) [M(Q)]	9.83 (7.00, 12.00)	8.00 (7.00, 14.00)	10.00 (7.00, 12.00)	−0.377	0.706^b^
Chemotherapy regimens (%)					
ECP/ECT	63 (58.3)	31 (57.1)	32 (59.6)	1.060	0.832^c^
ECTH/ECTHP	16 (14.8)	7 (12.5)	9 (17.3)		
TC	7 (6.5)	4 (7.1)	3 (5.8)		
TCbH/TCbHP	22 (20.4)	9 (23.2)	13 (17.3)		

^a^ Independent sample test.

^b^ Mann–Whitney U‐test.

^c^ Chi‐square test.

### 3.2 Comparison of the incidence of upper limb lymphedema after intervention

The results showed no significant difference in the incidence of lymphedema in the affected arm between the groups at T1 and T2 (*p* > 0.05, Table 2).

**TABLE 2:** Comparison of upper limb lymphedema incidence between the two groups after intervention.

Lymphedema	Post‐test 1		*χ* ^*2*^	*p*	Post‐test 2		*χ* ^*2*^	*p*
	Intervention (*n* = 52)	Control (*n* = 56)			Intervention (*n* = 52)	Control (*n* = 56)		
None (%)	51 (98.1)	54 (96.4)	0.000	1.000	50 (96.2)	52 (92.9)	0.107	0.744
Total (%)	1 (1.9)	2 (3.6)			2 (3.8)	4 (7.1)		

*Note*: *χ* ^2^, Chi‐square test.

### 3.3 Comparison of handgrip strength on the affected side after intervention between the two groups

The ANOVA results showed that the handgrip strength had a significant time effect (*F* = 268.147, *p* < 0.001) and interaction effect of group × time (*F* = 25.340, *p* < 0.001) during the study period, but the group effect was not significant (*F* = 2.434, *p* > 0.05). Further, comparisons between the two groups at the same time point found statistically significant differences in T1 (*t* = −2.512, *p* < 0.05) and T2 (*t* = −2.538, *p* < 0.05) scores between the two groups (Table 3).

**TABLE 3:** Comparison of handgrip strength between the two groups after intervention.

Group	Pre‐test $\bar{x}$ ± SD	Post‐test 1 $\bar{x}$ ± SD	Post‐test 2 $\bar{x}$ ± SD	Group	Time	Group × time	*η*P^2^ *
				*F* (*p*)	*F* (*p*)	*F* (*p*)	
Intervention	21.72 ± 5.58	18.40 ± 5.48	21.48 ± 5.36	2.434 (0.122)	268.147 (<0.001)	25.340 (<0.001)	0.325
Control	22.99 ± 5.08	16.12 ± 3.94	19.17 ± 4.04				
*t*	0.259	−2.512	−2.538				
*p*	0.796	0.014	0.013				

*Note*: *t*, independent sample test.

* The effect size of the interact effect of group × time.

### 3.4 Comparison of ROM on the affected side after the intervention in the two groups

**FIGURE 2:**
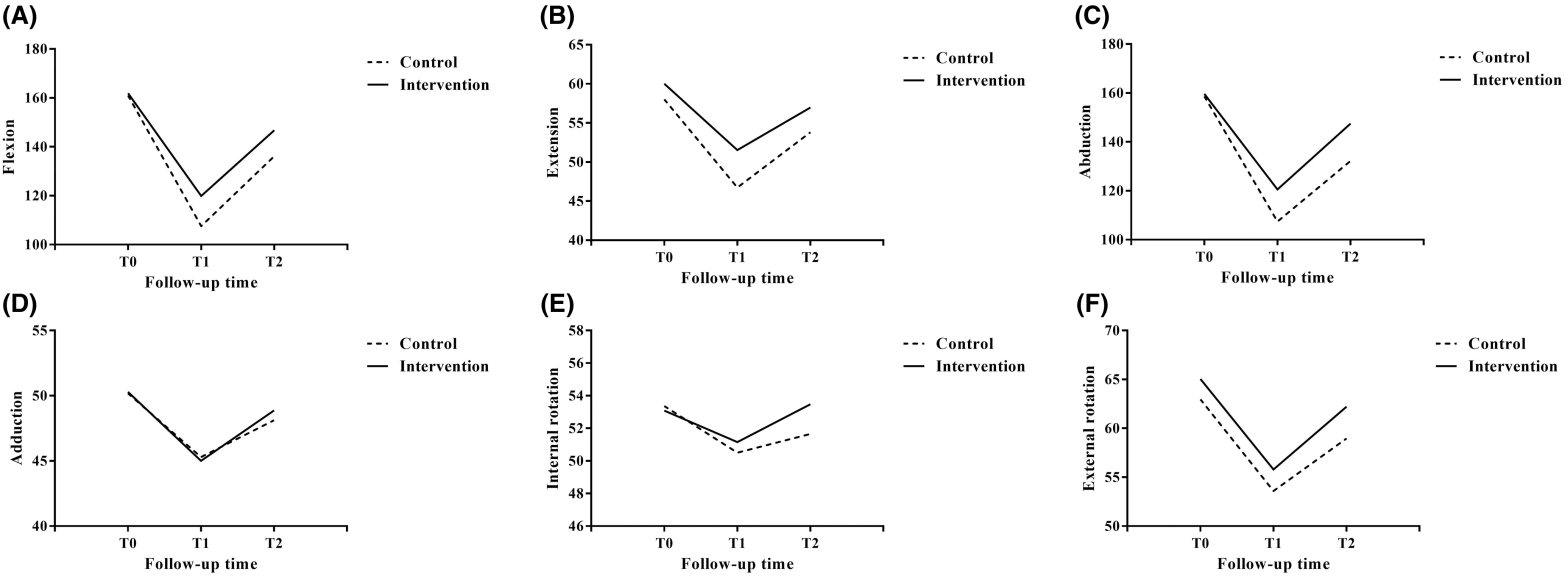
Comparison of ROM scores between the two groups after intervention.

Compared with the control group, the upper arm flexion and abduction scores were significantly higher for T1 and T2 in the intervention group. The T1 extension scores in the intervention group were significantly higher than the control group, while no significant changes were found in other ROM scores (Figure 2).

### 3.5 Comparison of DASH scores on the affected side after the intervention between the two groups

The ANOVA results showed a significant time effect, group effect, and interaction effect of group × time on the DASH scores of the affected side in the intervention and control groups. Compared between the two groups at the same time point, the DASH score of the affected side at T1 and T2 was both statistically significant (*p* < 0.05) (Table 4).

**TABLE 4:** Comparison of DASH scores between two groups after intervention.

Group	Pre‐test $\bar{x}$ ± SD	Post‐test 1 $\bar{x}$ ± SD	Post‐test 2 $\bar{x}$ ± SD	Group	Time	Group × time	*η*P^2^ *
				*F* (*p*)	*F* (*p*)	*F* (*p*)	
Intervention	3.03 ± 4.22	29.84 ± 11.85	11.65 ± 6.37	23.248 (<0.001)	367.612 (<0.001)	14.833 (<0.001)	0.220
Control	3.19 ± 3.19	38.00 ± 15.28	18.23 ± 6.27				
*t*	0.189	3.087	5.399				
*p*	0.851	0.003	<0.001				

*Note*: *t*, independent sample test.

* The effect size of the interact effect of group × time.

### 3.6 Comparison of FACT‐B scores on the affected side after the intervention between the two groups

**FIGURE 3:**
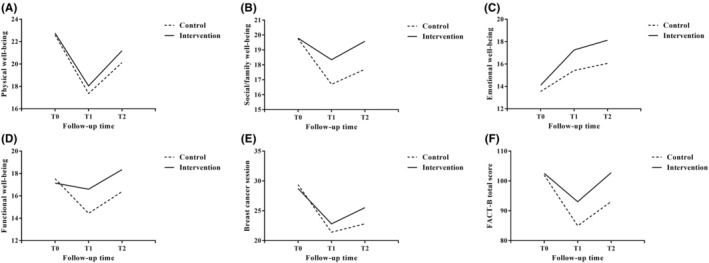
Comparison of FACT‐B scores between two groups after intervention.

Comparison between the two groups at T1 showed that the social/family well‐being, functional well‐being, and total scores of the patients in the intervention group were significantly higher than those in the control group (*p* < 0.05). At T2, the intervention group had higher scores in all dimensions than the control group, and except for physical well‐being, all other dimensions and total scores were statistically different from the control group (*p* < 0.05, Figure 3).

## 4 DISCUSSION

According to KAP theory, people's behavior change first needs to acquire relevant theoretical knowledge, then comes the attitude toward a certain behavior, and finally forms the behavior.^13^ This study followed the basic steps of KAP and developed a lymphedema prevention program. First, various health education methods were used to enhance patients' lymphedema‐related knowledge. Then, the hazards of lymphedema were emphasized and sharing the experiences of volunteers was to improve patients' attention to lymphedema. Finally, patients were urged to implement lymphedema prevention measures. This intervention based on KAP theory has been shown to be suitable for patient rehabilitation guidance and health education. Besides, this study also improved arm function and quality of life in patients with breast cancer. However, it did not have the expected effect on lymphedema incidence, possibly due to insufficient follow‐up time.

In this study, a lymphedema prevention program was implemented on breast cancer patients during the perioperative period and chemotherapy and was evaluated at T1 and T2. The results showed that fewer cases of lymphedema occurred in the intervention group than in the control group in both phases, but the incidence of lymphedema between the two groups was not statistically significant. In a study by Ammitzbøll et al.^27^ their intervention group performed progressive resistance training and self‐management exercises and their results also showed that exercise did not prevent upper extremity lymphedema but confirmed the importance and safety of resistance training for the patients. Similarly, another study that included 547 breast cancer patients who were intervened using a combination of lymphedema prevention or education plus exercise and physical therapy found no significant difference in the prevention of lymphedema despite interventions 18 months after surgery.^28^ However, a review that evaluated the effectiveness of prospective surveillance and early management of cancer‐associated lymphedema found that participation in prospective surveillance and early management reduced the risk of chronic BCRL,^29^ which differs from the findings of our study.

In a study comprising 2171 women with a median follow‐up of 4 years after breast cancer surgery, the investigators reported that the risk of lymphedema peaked between 12 and 30 months after surgery, with the incidence of lymphedema peaking between 6 and 12 months in patients with lymphatic dissection.^30^ Since the outcome indicators in this study needed to be measured by professionals to ensure the accuracy of outcome measurements and patient compliance, considering that the COVID‐19 affected patients' travel, the outcome indicators were measured when patients' returned for treatment. T2 was performed at the patient's 6th chemotherapy visit. The duration of one chemotherapy cycle was 21 days, and the 6th postoperative visit was equivalent to 18 weeks after surgery. Therefore, it is also possible that the negative result was due to the short follow‐up period. However, upper limb lymphedema occurred in both groups in this study, which indicated that BCRL occurred at an early stage (within 4 months) in some patients, suggesting the need for focusing on early prevention and evaluation of early detection and treatment of BCRL.^10^ This study found that implementing preventive measures and nursing interventions related to lymphedema were safe and beneficial, but the effectiveness of prevention of lymphedema should be followed up for a longer time in future studies.

After surgery, the handgrip strength of the affected upper limb decreased significantly in both groups compared to the preoperative period, similar to the results reported by Klassen et al.^31^ who reported that the upper extremity muscle strength in patients with breast cancer during postoperative treatment was reduced by 12% to 16% compared to healthy individuals. On the one hand, it could be due to the impact of surgery on the muscle damage of the upper limbs, while on the other hand, the wound pain of the patients after the surgery might have reduced activities of the upper limbs, which led to muscle atrophy. Our implemented lymphedema prevention program included rehabilitation exercises, aerobic exercise, and resistance training. A meta‐analysis of 29 studies on the effects of resistance exercise on lymphedema and muscle strength and pooled strength results from six studies showed significant improvements in both upper and lower extremity muscle strength in patients.^32^ In this study, the results of handgrip strength of the upper limbs on the affected side of the two groups of patients at T1 and T2 were *p* < 0.05, which was consistent with the findings of Hasenoehrl,^32^ who indicated that this prevention program could enhance upper limb muscle function and improve the handgrip strength of the affected arm in patients after breast cancer surgery.

In this study, the range of motion of the upper limb and the DASH score of the two groups of patients were in the normal range before the intervention. As breast cancer patients have significant muscle damage and joint dysfunction during treatment,^31^ we observed a significant time effect for the range of motion of the upper limb and DASH scores on the affected side in both intervention and control patients. Although the ROM of flexion, extension and abduction were statistically different between the two groups, there was no significant difference in the ROM of adduction and external rotation at T1. One study reported ROM flexion and abduction deficits in breast cancer patients at 2 months after surgery, with partial recovery at 30 months postoperatively,^33^ similar to the study's results. Additionally, although there were also statistically significant differences in flexion and abduction between the two groups, there was no statistical difference in the range of motion in other directions at T2. Therefore, this preventive program significantly improved the flexion and abduction of the affected shoulder joint in patients after surgery, and the impact on the extension of the shoulder joint was mainly reflected in the early postoperative period.

At T1 and T2, the DASH scores significantly decreased in both the control and intervention groups. Previous studies found that postoperative exercise improved shoulder function in women at higher risk of shoulder problems after breast cancer surgery. A study using a nurse‐led activity program and MLD to prevent BCRL showed that both measures were effective and improved arm function.^34^ Zhang et al. randomly divided 300 perioperative breast cancer patients into a control group (underwent routine care) and an intervention group (comprised of instructions for patients to learn about damage recognition before surgery, self‐monitoring, self‐management, and health promotion). Their result showed that 3 months after surgery, the total DASH scores of the two groups were higher than those before surgery and the scores of the intervention group and the control group were statistically different, which was similar to the results of this study.^35^ Since the intervention programs in this study included functional exercise and nurse‐led activity programs, and the DASH scores of both groups were *p* < 0.05 at the two evaluations, these findings showed that the implemented prevention program could significantly improve upper limb dysfunction in breast cancer patients after surgery.

Advances in early detection and treatment can increase patient survival after breast cancer diagnosis, which has also prompted more women to live with the consequences of cancer treatment, rendering the quality of life of patients after breast cancer surgery to become a major concern. Biparva et al. conducted a meta‐analysis on 9012 BC patients and found that the quality of life scores of the patients who completed the treatment were higher than patients who were still being treated,^36^ consistent with the findings of our study, which showed that both groups had improved quality of life 18 weeks after surgery compared to the earlier postoperative periods. At T1, the control group and the intervention group had statistically different social/family well‐being, functional well‐being, and total scores, indicating that the program could promote the recovery of the patients' functional state and accelerate the return to normal family and social well‐being. At T2, the scores of the two groups were statistically significant except for the physical well‐being scores, similar to the results of Omidi et al.^37^ Current evidence suggests that educating the patients and providing specialized breast disease care services and social support from institutions could help reduce unmet needs and improve the patients' quality of life.^38^ Thus, the lymphedema prevention program seems promising in promoting physical and mental recovery and improving the quality of life of breast cancer patients.

## 5 LIMITATIONS

There are some limitations in this study. On the one hand, the subjects in this study were limited to breast cancer patients receiving treatment in one hospital. The sampling range and sample size were limited. In addition, due to time constraints, this study only evaluated the effect of intervention for 4 months without continuous follow‐up investigation, and the long‐term effects of lymphedema prevention interventions cannot be judged. As lymphedema is a secondary complication, the incidence may increase with the influence of time or radiotherapy after chemotherapy, so the negative result may be caused by the short follow‐up time in this study.

## 6 CONCLUSION

Overall, this study provides some evidence for the intervention effectiveness of KAP theory. The knowledge, attitude, and behavior of the KAP theory were combined in the perioperative period and the first three times of chemotherapy for breast cancer patients. First of all, we aim to improve the cognition of breast cancer patients to lymphedema; then, we focus on improving their attention to lymphedema. In addition finally, we could promote their clinical edema prevention behavior. This RCT found that although the lymphedema prevention program could not reduce the incidence of lymphedema in early postoperative breast cancer patients, it could significantly improve their arm function and quality of life. Follow‐up could be extended in the future to further evaluate the effectiveness of the lymphedema prevention program.

## AUTHOR CONTRIBUTIONS

**Bohui Shi:** Conceptualization (lead); data curation (equal); methodology (lead); project administration (lead); resources (equal); software (equal); supervision (equal); validation (equal); visualization (equal); writing – original draft (lead); writing – review and editing (equal). **Zihan Lin:** Data curation (lead); investigation (lead); project administration (supporting); writing – original draft (supporting). **Xiaowei Shi:** Conceptualization (equal); formal analysis (equal); methodology (lead); project administration (equal); resources (equal); supervision (equal); writing – review and editing (supporting). **Pingli Guo:** Project administration (equal); resources (lead); supervision (equal). **Wen Wang:** Data curation (lead); investigation (equal); validation (equal); writing – review and editing (supporting). **Xin Qi:** Writing – review and editing (equal). **Can Zhou:** Project administration (equal); resources (lead); supervision (equal). **Huifang Zhang:** Data curation (equal); investigation (lead); software (equal). **Xiaona Liu:** Methodology (lead); resources (supporting). **Aili Iv:** Conceptualization (lead); methodology (equal); project administration (lead); resources (supporting); supervision (lead); validation (equal); writing – original draft (supporting); writing – review and editing (lead).

## FUNDING INFORMATION

Funding was provided by the Key research and development project of Shaanxi Province (Grant No: 2019SF‐145).

## CONFLICT OF INTEREST STATEMENT

The authors declare no conflicts of interest.

## Data Availability

The data that support the findings of this study are available from the corresponding author upon reasonable request.

## APPENDIX 1

### Intervention plan for two groups of patients.

**Table:**

Time	Control group	Intervention group
Perioperative	Routine preoperative education: diet, blood pressure, psychological guidance, skin preparation, etc.	Routine preoperative education; Introduction to common postoperative complications.
	Routine postoperative education: body position, diet, drainage tube, psychology, wound care and guiding the patients to perform early functional exercises.	Routine nursing; Patients and their families were organized to learn the specialty content in the recovery room, with a 30‐min PPT lecture by a physician member of the subject group. The content was based on the definition, type, mechanism of occurrence, risk factors, grading, and incidence of upper limb lymphedema in postoperative breast cancer; A 30‐min seminar after the lecture: the panelists assessed the knowledge of the patients and their families about lymphedema and answered questions.
	Routine health education: inform patients about rehabilitation exercises and 18 lymphedema risk management measures in the ward display board.	Routine health education; Information on the best evidence for preventing upper extremity lymphedema after breast cancer surgery was given to each patient (exercise methods, skin protection, air travel, weight control, physical therapy, self‐monitoring and daily life precautions, 7 dimensions, and a total of 48 preventive measures).
	Routine discharge education: regular medication change, review and incoming chemotherapy.	Routine discharge education; Diary cards for exercise and avoidance of behaviors that could increase the risk of lymphedema were given and instructed to fill out daily; Instructed the patients and family members to follow the department's official WeChat account and learned to view the content of health education and postoperative functional exercise exercises at different stages; Established a WeChat group. Published the official account content regularly and timely answered questions in the WeChat group.
	Routine discharge follow‐up: telephone to ask patients about their wounds, drainage, activities and discomfort.	Routine discharge follow‐up; Enquired about the exercise and presence of any swelling and discomfort and provided guidance.
First chemotherapy cycle	Routine admission examinations: electrocardiogram, blood routine, etc.	Routine admission examinations; Reviewed diary cards and provided guidance and education.
	Routine nursing: chemotherapy side effects care and health education.	Routine nursing; Patients and their families were organized to learn the specialty content in the recovery room, with a 30‐min PPT lecture by a nurse who was trained on the lymphedema training course of the subject group. The content comprised early recognition of upper limb lymphedema, assessment methods, common treatment methods and treatment institutions; A 30‐min seminar after the lecture: to communicate with patients and families about upper extremity lymphedema and their knowledge of prevention. Assist patients in analyzing potential bad habits, recognizing the dangers of unhealthy behaviors, and discussing correction plans. Provided psychological guidance to patients and family members who were blindly optimistic or overly worried; Played functional exercise videos in the recovery room, urged patients to follow the exercises and instructed them to standardize their movements.

	Patient communication	Patient communication; Rehabilitation volunteers (two at a time) come to the ward to form mutual help groups with patients under the guidance of subject team members. Sharing their own rehabilitation experiences, transmitting positive energy, increasing patients’ confidence in the recovery, and instructing them on precautions and exercise methods in their lives; Encourage each other and share experiences among patients in the WeChat group, with the administrator participating in the interaction and offering professional timely guidance.
	Routine discharge education: regular blood tests and returning to the hospital for chemotherapy.	Routine discharge education; Instruct regular exercise and avoid behaviors that can lead to lymphedema. Emphasis on filling out diary cards.
Routine discharge follow‐up: telephone to ask patients about their wounds, drainage, activities and discomfort.	Routine discharge follow‐up; Enquiry on the exercise and presence of any swelling and discomfort and provided guidance.
Second chemotherapy cycle	Routine admission examinations: electrocardiogram, blood routine tests, etc.	Same as 1st chemotherapy
	Routine nursing: chemotherapy side effects care and health education.	Routine nursing; Patients and their families were organized to learn about the specialty content in the recovery room, with a 30‐min PPT lecture by a lymphedema technician of the subject group. The content was based on the consequential effects of upper limb lymphedema and preventive measures; A 30‐min seminar after the lecture: to instruct the patients and families on early recognition of lymphedema and perform assessment methods; Played functional exercise videos in the recovery room, urged patients to follow the exercises and instructed them to standardize their movements.
	Patient communication	Same as 1st chemotherapy
	Routine discharge education	
	Routine discharge follow‐up	
Third chemotherapy cycle	Routine admission examinations: electrocardiogram, blood routine tests, etc.	Same as 1st chemotherapy
	Routine nursing: chemotherapy side effects care and health education.	Routine nursing; Assessed patients’ knowledge of lymphedema prevention and exercise compliance and continuously provided education and guidance; Played functional exercise videos in the recovery room, urged patients to follow the exercises, and instructed them to standardize their movements.
	Patient communication	Same as 1st chemotherapy
	Routine discharge education	
	Routine discharge follow‐up	

## References

[1] Sung H , Ferlay J , Siegel RL , et al. Global cancer statistics 2020: GLOBOCAN estimates of incidence and mortality worldwide for 36 cancers in 185 countries. CA Cancer J Clin. 2021;71:209‐249. 33538338 10.3322/caac.21660

[2] Al‐Hilli Z , Wilkerson A . Breast surgery: management of postoperative complications following operations for breast cancer. Surg Clin North Am. 2021;101:845‐863. 34537147 10.1016/j.suc.2021.06.014

[3] Azhar SH , Lim HY , Tan B‐K , Angeli V . The unresolved pathophysiology of lymphedema. Front Physiol. 2020;11:137. 32256375 10.3389/fphys.2020.00137 PMC7090140

[4] Jørgensen MG , Toyserkani NM , Hansen FG , Bygum A , Sørensen JA . The impact of lymphedema on health‐related quality of life up to 10 years after breast cancer treatment. NPJ Breast Cancer. 2021;7:70. 34075045 10.1038/s41523-021-00276-y PMC8169644

[5] DiSipio T , Rye S , Newman B , Hayes S . Incidence of unilateral arm lymphoedema after breast cancer: a systematic review and meta‐analysis. Lancet. 2013;14:500‐515. 10.1016/S1470-2045(13)70076-7 23540561

[6] Marchica P , D'Arpa S , Magno S , et al. Integrated treatment of breast cancer‐related lymphedema: a descriptive review of the state of the art. Anticancer Res. 2021;41:3233‐3246. 34230117 10.21873/anticanres.15109

[7] Sun W , Liu H , Dong L , Sun R , Guo L , Zhang H . Cognition of postoperative lymphedema among breast cancer patients in Lianyungang area. Chinese J Clin Res. 2020;33:856‐859.

[8] Kwan ML , Shen L , Munneke JR , et al. Patient awareness and knowledge of breast cancer‐related lymphedema in a large, integrated health care delivery system. Breast Cancer Res Treat. 2012;135:591‐602. 22903688 10.1007/s10549-012-2199-x

[9] Zhao H , Wu Y , Zhou C , Li W , Li X , Chen L . Breast cancer‐related lymphedema patient and healthcare professional experiences in lymphedema self‐management: a qualitative study. Support Care Cancer. 2021;29:8027‐8044. 34226959 10.1007/s00520-021-06390-8

[10] Whitworth P , Vicini F , Valente SA , et al. Reducing rates of chronic breast cancer‐related lymphedema with screening and early intervention: an update of recent data. J Cancer Surviv. 2022; Online ahead of print. 10.1007/s11764-022-01242-8 35947288

[11] Ridner SH , Fu MR , Wanchai A , Stewart BR , Armer JM , Cormier JN . Self‐management of lymphedema: a systematic review of the literature from 2004 to 2011. Nurs Res. 2012;61:291‐299. 22565103 10.1097/NNR.0b013e31824f82b2

[12] Fu X , Lu Q , Pang D , Shen A , Shih Y‐A , Wei X . Experiences of breast cancer survivors with lymphedema self‐management: a systematic review of qualitative studies. J Cancer Surviv. 2023;17(3):619‐633. 35773611 10.1007/s11764-022-01225-9

[13] Fan Y , Zhang S , Li Y , et al. Development and psychometric testing of the knowledge, attitudes and practices(KAP)questionnaire among student tuberculosis(TB)patients(STBP‐KAPQ)in China. BMC Infect Dis. 2018;18:213. 29739363 10.1186/s12879-018-3122-9 PMC5941627

[14] Lee M , Kang B‐A , You M . Knowledge, attitudes, and practices (KAP) toward COVID‐19: a cross‐sectional study in South Korea. BMC Public Health. 2021;21:295. 33546644 10.1186/s12889-021-10285-y PMC7863060

[15] Bihon A , Zinabu S , Muktar Y , Assefa A . Human and bovine tuberculosis knowledge, attitude and practice (KAP) among cattle owners in Ethiopia. Heliyon. 2021;7:e06325. 33748453 10.1016/j.heliyon.2021.e06325 PMC7969333

[16] Wang J , Chen L , Yu M , He J . Impact of knowledge, attitude, and practice (KAP)‐based rehabilitation education on the KAP of patients with intervertebral disc herniation. Ann Palliative Med. 2020;9:388‐393. 10.21037/apm.2020.03.01 32233633

[17] Yue T , Zhuang D , Zhou P , et al. A prospective study to assess the feasibility of axillary reverse mapping and evaluate its effect on preventing lymphedema in breast cancer patients. Clin Breast Cancer. 2015;15:301‐306. 25776198 10.1016/j.clbc.2015.01.010

[18] Yunjuan G , Xiaomin Z , Yali Z . Application of comfort nursing intervention in preventing postoperative lymph edema in patients undergoing radical mastectomy for breast cancer. Int J Nurs. 2020;39:4536‐4539.

[19] Shi B ; Lv A , Wang L , Guo P , Ma X , Qi J . Evidence summary for prevention strategies of breast cancer‐related lymphedema. J Nurs. 2020;27:32‐38.

[20] Wang Y , Qiang W . Evaluation and management of breast cancer associated lymphedema. Tianjin J Nurs. 2017;24:366‐368.

[21] Maldonado GEM , Pérez CAA , Covarrubias EEA , et al. Autologous stem cells for the treatment of post‐mastectomy lymphedema: a pilot study. Cytotherapy. 2011;13:1249‐1255. 21999374 10.3109/14653249.2011.594791

[22] Park J‐H . The effects of complex exercise on shoulder range of motion and pain for women with breast cancer‐related lymphedema: a single‐blind, randomized controlled trial. Breast Cancer. 2017;24:608‐614. 28008557 10.1007/s12282-016-0747-7

[23] Beaton DE , Wright JG , Katz JN ; Group UEC . Development of the QuickDASH: comparison of three item‐reduction approaches. J Bone Joint Surg. 2005;87:1038‐1046. 15866967 10.2106/JBJS.D.02060

[24] Liao C , Wang C , Zhou X , Wang X . Checkout reliability and validity of Chinese version of DASH short form scale applied in upper limb dysfunction evaluation research of breast cancer patient. Chin Nurs Res. 2014;28:3581‐3583.

[25] Cella DF , Tulsky DS , Gray G , et al. The functional assessment of cancer therapy scale: development and validation of the general measure. J Clin Oncol. 1993;11:570‐579. 8445433 10.1200/JCO.1993.11.3.570

[26] Wan C , Zhang D , Tang X , et al. Revision of the Chinese version of the FACT‐B for patients with breast cancer. Chin Ment Health J. 2003;17:298‐300.

[27] Ammitzbøll G , Johansen C , Lanng C , et al. Progressive resistance training to prevent arm lymphedema in the first year after breast cancer surgery: results of a randomized controlled trial. Cancer. 2019;125:1683‐1692. 30633334 10.1002/cncr.31962

[28] Naughton MJ , Liu H , Seisler DK , et al. Health‐related quality of life outcomes for the LEAP study‐CALGB 70305 (Alliance): A lymphedema prevention intervention trial for newly diagnosed breast cancer patients. Cancer. 2020;127:300‐309. 33079393 10.1002/cncr.33184 PMC7790999

[29] Rafn BS , Christensen J , Larsen A , Bloomquist K . Prospective surveillance for breast cancer‐related arm lymphedema: a systematic review and meta‐analysis. J Clin Oncol. 2022;40:1009‐1026. 35077194 10.1200/JCO.21.01681

[30] McDuff SGR , Mina AI , Brunelle CL , et al. Timing of lymphedema after treatment for breast cancer: when are patients Most At risk? Int J Radiat Oncol Biol Phys. 2019;103:62‐70. 30165125 10.1016/j.ijrobp.2018.08.036 PMC6524147

[31] Klassen O , Schmidt ME , Ulrich CM , et al. Muscle strength in breast cancer patients receiving different treatment regimes. J Cachexia Sarcopenia Muscle. 2017;8:305‐316. 27896952 10.1002/jcsm.12165 PMC5377413

[32] Hasenoehrl T , Palma S , Ramazanova D , et al. Resistance exercise and breast cancer‐related lymphedema‐a systematic review update and meta‐analysis. Support Care Cancer. 2020;28:3593‐3603. 32415386 10.1007/s00520-020-05521-x PMC7316683

[33] Oliveira MMF , Gurgel MSC , Amorim BJ , et al. Long term effects of manual lymphatic drainage and active exercises on physical morbidities, lymphoscintigraphy parameters and lymphedema formation in patients operated due to breast cancer: a clinical trial. PLoS One. 2018;13:e0189176. 29304140 10.1371/journal.pone.0189176 PMC5755747

[34] Dönmez AA , Kapucu S . The effectiveness of a clinical and home‐based physical activity program and simple lymphatic drainage in the prevention of breast cancer‐related lymphedema: a prospective randomized controlled study. Eur J Oncol Nurs. 2017;31:12‐21. 29173822 10.1016/j.ejon.2017.09.004

[35] Zhang H , Duan Y , Zhou F . Explore the application value of prospective monitoring model in the nursing management of breast cancer patients during perioperative period. Front Surg. 2022;9:850662. 35284475 10.3389/fsurg.2022.850662 PMC8906514

[36] Biparva AJ , Raoofi S , Rafiei S , et al. Global quality of life in breast cancer: systematic review and meta‐analysis. BMJ Support Palliat Care. 2022. doi:10.1136/bmjspcare-2022-00364 PMC10850719 35710706

[37] Omidi Z , Kheirkhah M , Abolghasemi J , Haghighat S . Effect of lymphedema self‐management group‐based education compared with social network‐based education on quality of life and fear of cancer recurrence in women with breast cancer: a randomized controlled clinical trial. Qual Life Res. 2020;29:1789‐1800. 32152817 10.1007/s11136-020-02455-z PMC7295820

[38] Ho PJ , Gernaat SAM , Hartman M , Verkooijen HM . Health‐related quality of life in Asian patients with breast cancer: a systematic review. BMJ Open. 2018;8:e020512. 10.1136/bmjopen-2017-020512 PMC5914715 29678980

